# The Rise of an Oppurtunistic Infection called “Invasive Zygomycosis”

**DOI:** 10.4103/0974-777X.56256

**Published:** 2009

**Authors:** Abdelkarim Waness, Ghuzayel Al Dawsari, Hamdan Al Jahdali

**Affiliations:** *Department of Internal Medicine, King Abdulaziz Medical City, Riyadh, Saudi Arabia*; 1 *Department of Hematology, King Abdulaziz Medical City, Riyadh, Saudi Arabia*; 2 *Department of Pulmonology, King Abdulaziz Medical City, Riyadh, Saudi Arabia*

**Keywords:** Pulmonary mucormycosis, Diabetes mellitus, Aplastic anemia, Amphotericin B, Death

## Abstract

Invasive zygomycosis is a devastating fungal infection seen mostly in immune-compromised patients. We present a case of a 48-year old diabetic man, with aplastic anemia, who developed severe pulmonary mucormycosis that led to his rapid demise despite early diagnosis and treatment with liposomal amphotericin B. We also conducted an extensive review of the pathogenesis of invasive zygomycosis, its history, predisposing factors, clinical aspects, diagnostic modalities, treatment options, morbidity and mortality.

## INTRODUCTION

Zygomycosis, also known as mucormycosis or phycomycosis or hyphomycosis, is a rapidly-progressive life-threatening deep fungal infection primarily affecting patients with decreased immunity. Rare compared to other infectious pathologies, it is gaining more ground recently. Mucormycosis has predilection for certain groups of people, including immune-suppressed and diabetic patients. This aggressive infection comes in a variety of forms. Despite recent advances in its medical and surgical treatments, it still retains a poor prognosis with high morbidity and mortality.

## CASE PRESENTATION

**Figure 1:**
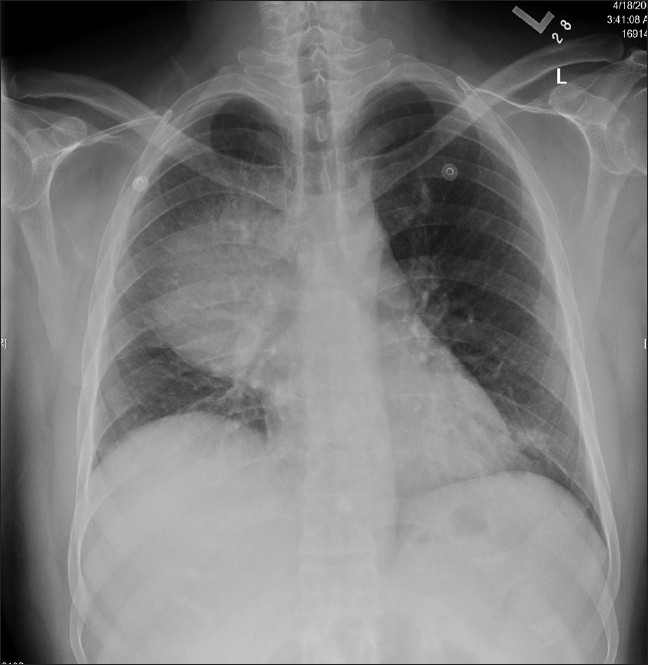
Admission chest X-ray showing large consolidation involving the right upper lobe

**Figure 2:**
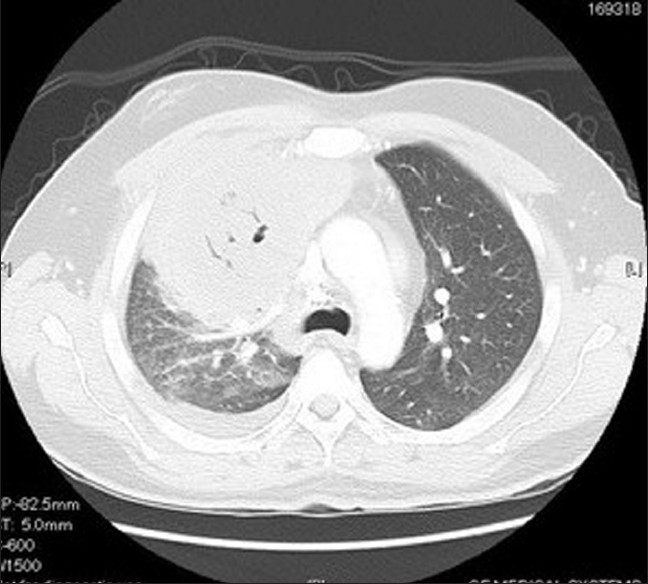
Computed tomography of the chest showing large mass with central hypodensity involving right upper lobe

**Figure 3:**
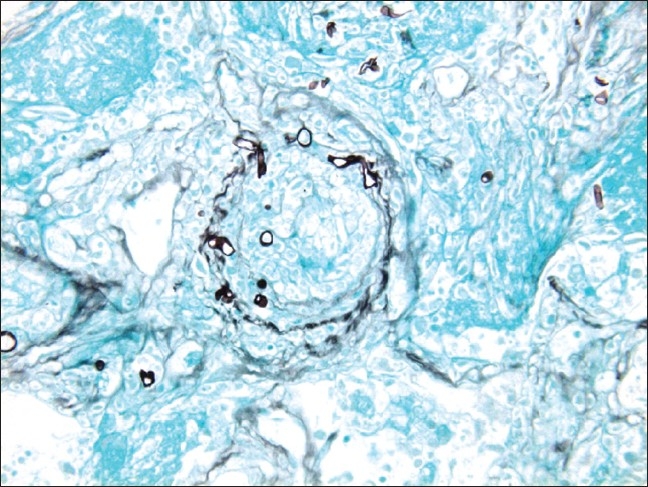
Histopathology of the right lung biopsy showing short broad fungal hyphae with vascular obliteration (GMS stain)

A 48-year-old non-smoking male, with past medical history of diabetes mellitus and aplastic anemia, was admitted to our hospital with fever, substernal chest pain and dyspnea of three-week duration. On examination, he was in mild respiratory distress. His vital signs were: temperature 38.8° C, pulse 92 bpm, respiratory rate 24 per minute, BP 115 / 64 mm Hg, and oxygen saturation of 95% on room air. He had right-sided chest dullness and crackles. The rest of his exam was negative. His medication included insulin and tacrolimus: 1 mg orally twice a day. His laboratory findings were: WBC 0.5×10^6^/L, hemoglobin 79 g/l, platelets 9×10^6^/L, glucose 13.7 mmol/l, sodium 125 mmol/l, bicarbonate 13 mmol/l, serum creatinine 143 micromol/l, INR 1.5, PT 13.3 sec. Cultures, including acid-fast bacilli, were negative. On his admission, chest x-ray showed a large round opacity adjacent to the right hilum [Figure 1]. Computerized Tomography (CT) scan of the chest confirmed the presence of a 9 × 9 cm mass, with air bronchograms, occupying most of the right upper lobe [Figure 2]. He was admitted with the working diagnosis of neutropenic fever and right upper lobe lung mass. He was started on broad antibiotic coverage with ceftazidime, vancomycin and caspofungin. The patient underwent transthoracic core biopsy of this mass, the histopathologic diagnosis was: pulmonary mucormycosis [Figure 3]. He was subsequently switched to intravenous liposomal amphotericin B therapy. His condition continued to deteriorate requiring transfer to the Intensive Care Unit for mechanical ventilation. His blood count never recuperated despite treatment with granulocyte-colony stimulating factor (G-CSF) and repetitive transfusions. He died 12 days after admission.

## ETIOLOGY / PATHOGENESIS

Mucormycosis is a broad term for a multitude of diseases caused by infection with different fungi in the order of Mucorales. The most common causative organisms are from the *Rhizopus* species. Other species include, in descending order, include *Rhizomucor, Cunninghamella, Apophysomyces, Saksenaea, Absidia, Mucor, Syncephalastrum, Cokeromyces,* and *Mortierella.*[1] These fungi are ubiquitous in nature and have world-wide distribution. Mucor is a rapidly growing fungus that is usually dark gray or light olive gray when grown on typical laboratory media. It is easily recognizable microscopically by its tall needle like sporangiophores and large sporangium. The mold grows and spreads quickly. Like other members of the class Zygomycetes, Mucor fungi can reproduce asexually with spores, or sexually by fusing to create zygospores which contain a mixture of genetic material. Mucor can be present in the outdoor or indoor settings. In the outdoors, it can be found in soil, decaying vegetation, hay, stored seeds or horse manure. Indoors, it can be found in house dust, and poorly maintained vacuum systems or dirty carpets. One study looking at the most frequent molds found in house dust found Mucor in 98% of the samples from homes in Denmark and 31% of the samples in homes in Canada.[2] Heavy inhalation of the Mucor spores can cause extrinsic allergic alveolitis and ultimately pulmonary fibrosis if the fungus exposure persists. The supreme danger of Mucor, however, lays in the fact that it can become an opportunistic pathogen causing deep fungal infection when conditions are right. Ripe conditions for aggressive zygomycosis include significantly compromised immunity such in malignancy, neutropenia, use of immunosuppressive agents, metabolic acidosis, uncontrolled diabetes, starvation, severe trauma or other forms of debilitation. It is well documented that they can cause a multitude of pathologies not only in humans but also in cattle, sheep, swine and dogs.[3] In Australia, mucormycosis was documented to cause severe skin lesions in frogs[4] and the *Tasmanian platypus.[5]* The histopathologic hallmark of this infection is the mycotic invasion of the blood vessels, often leading to thrombosis, followed by tissue infarction and necrosis mediated by fungal proteases, lipases and mycotoxins.[6] This aggressive vasculature invasion can not only affect the small vessels such as arterioles but can also reach large arteries causing devastating results such as rupture of the aorta.[7]

## HISTORY / EPIDEMIOLOGY

It is likely that Mucormycosis had accompanied human existence since a long time. The first documented case, however, is relatively recent. In 1885, Paltauf pioneered the publication of a case of upper airway mucormycosis, entitled: “mucormycosis mucorina” in the Virchows archives of pathology and anatomy.[8] In 1943, Gregory and associates reported the more typical findings of advanced rhinocerebral mucormycosis in three patients with diabetic ketoacidosis.[9] Mucormycosis is certainly seen less than other common fungal infections like candidiasis or aspergillosis. However, its incidence has been increasing recently. Brown has reported that the frequency of zygomycosis has been increasing over the past 14 years in the United States of America; this fungal infection has been identified in up to 6.8% of patients at autopsy.[10] Another study confirmed that the incidence of this infection is also on the rise in Europe.[11] This rise is partially explained by better diagnostic tools, increased incidence of diabetes mellitus and use of immunosuppressive agents in the modern therapeutic era.[12]

## MORTALITY / MORBIDITY

Invasive zygomycosis is simply bad news for patients as well as treating physicians. Indeed, and despite recent medical advances, this aggressive fungal infection still carries poor prognosis. Since deep mucormycosis encompasses many syndromes, the mortality rate varies greatly from 33.3% in a Korean study,[13] to a worse rate of 63% in an Italian study,[14] to a staggering 96% rate in case of disseminated form.[15] This extreme variation in mucormycosis mortality rate can be explained by many factors, including early diagnosis, site of the infection, patient's immune status, correction of other co-morbid factors, and the type of therapy instituted among others.

If the patient, struck with aggressive zygomycosis, survives this horrible initial infection; he has high probability of carrying some of its terrible and severe debilitating consequences. Spontaneous blindness due to bilateral ophthalmic artery occlusion in rhino-orbito-cerebral mucormycosis has been reported.[16] Facial disfiguration is a common result of aggressive surgery in cases of rhino maxillary or orbital mucormycosis.[17] With aggressive pulmonary zygomycosis, complete pneumonectomy and even partial chest wall resection can be performed.[18] In the case of bilateral renal mucormycosis, both kidneys had to be removed to save the patient's life.[19] Obviously, these patients will need physical and psychological support and occasionally rehabilitation with their daily activities.

## GENDER / AGE

Three previous review studies, carried in 1971, 1994 and 1999, show an unexplained predilection of zygomycosis to the male gender. The male-female ratio was between 2.4:1 and 3:1.[20] This finding was confirmed by the 2005 larger review study done by Roden and associates: 65% of the reviewed cases were males.[15] The mean age in these previous review studies was in the 30s to 40s. However, the spectrum of age of patients suffering from this invasive mycosis is very wide: from neonates[21] to the very old.[16]

## PREDISPOSING FACTORS

Invasive zygomycosis can rarely occur in healthy individuals without any apparent predisposing factors.[22] It is usually a disease of the immune-compromised patient or those with chronic debilitating conditions. Some of these predisposing conditions include: ketoacidosis and uncontrolled diabetes mellitus,[23] other forms of acidosis,[24] hematologic malignancies[14] or solid cancers,[25–27] immunosuppressive therapy even for a short period of time,[28–30] after solid organ[31–33] or following bone marrow transplantation,[34] patients with congenital or acquired neutropenia[35] or anemia such as thalassemia or aplastic anemia.[36–37] Individuals with acquired immunodeficiency syndrome (AIDS) are also prone to invasive phycomycosis among other fungal infections,[38–39] persons with history of intravenous drug abuse[40] or history of alcoholism;[41] it can be seen following trauma[42–43] or with different degrees of burns.[44–45] Hospital acquired zygomycosis can occur following invasive procedures such as central line catheter or pace-maker wire implantation[46–47] or frank surgical intervention.[48–49] Other potential predisposing factors include chronic infections such as tuberculosis,[50] septicemia with multi-organ failure,[51] fever of unknown origin,[52] use of antibiotic therapy, chronic renal failure,[53] sarcoidosis,[54] or patients suffering from starvation and severe malnutrition.[55] Iron metabolism is another important predisposing factor for invasive zygomycosis. Even partially understood, many studies demonstrated that increased serum iron and treatment with the iron chelating agent deferoxamine predispose to such condition. The Rhizopus species actually utilize deferoxamine as a siderophore to supply previously unavailable iron to the fungus; this increased iron uptake is linearly correlated with its growth in the serum.[56–57] The list of potential predisposing factors for invasive zygomycosis will certainly keep on growing with the advent of new biologic therapeutic agents that adversely affect patients' immunity.

## MAJOR CLINICAL ZYGOMYCOSIS SYNDROMES

Classically, invasive zygomycosis has been classified into six different clinical syndromes. This classification is based on the general location of the disease. These locations are: rhino-cerebral, pulmonary, gastro-intestinal, cutanous, disseminated and miscellaneous. We will adopt this classification for the lack of a better one, but keep in mind the wide variety of combinations and presentations that can be adopted by mucormycosis.

Rhino-cerebral zygomycosis: It is the most common form of all invasive mucormycoses form with one third to half of all cases.[56] It is seen primarily in uncontrolled diabetic patients. It occurs by inhalation or hematogenous or lymphatic dissemination. The classical form involves the sinuses, especially maxillary, but can easily spread to the neighboring tissue including nose, orbits, eyes, brain, cranial nerves, hard and soft palates, both mandibles and the rest of the face. It has great variety of clinical presentations from that of a simple acute sinusitis with purulent rhinorrhea,[58] where the initial exam of the nasal mucosa may be normal, to a more dramatic presentation caused by progressive thrombosis and infarction. The exam might reveal violaceous discoloration, black eschar or frank tissue necrosis. Fever can be present or absent. Another well-recognized and severe form of rhino-cerebral zygomycosis is the presentation of periorbital cellulitis. It can be uni or bilateral. Initially it presents with tissue edema and erythema around the eye(s), later proptosis, ophtalmoplegia, and visual loss can ensue.[59] The spread of the infection to the hard palate can cause perforation.[60] Its extension towards the brain can cause utmost devastation; cases with brain abscesses,[61] cerebral arteries aneurysms,[62] hydrocephalus[63] and stroke[64] have been reported. Even the rare Garcin syndrome, where multiple cranial neuropathies occur, was documented in mucormycosis.[65]

Pulmonary mucormycosis: It is thought to be second most common form. It occurs by inhalation or hematogenous or lymphatic spread. It can present with mild to severe symptoms including fever, cough, sputum production, dyspnea, hypoxia, chest pain and hemoptysis.[64] Mucormycosis can cause lobar consolidation,[66] multiple disseminated lung nodules,[67] fungal ball[68] or mycotic abcess formation.[69] It can also present as a single tracheal[70] or multiple endobronchial lesions.[71] Obviously, and because of its proximity, pulmonary zygomycosis can disseminate to the pleural space,[72] chest wall,[73] or the mediastinum where it can cause catastrophic rupture of large vessels.[7]

Gastrointestinal zygomycosis: It is relatively rare. It is thought to be caused by ingestion of zygospores especially in the malnourished and alcoholics,[74] or it can be secondary to trauma.[75] It can cause fever, abdominal pain and bloating, nausea and vomiting, hematemesis, melena or bowel perforation. It can be observed in the stomach where it can cause ulceration, bleeding or perforation.[76] It is interesting to notice that cases of iatrogenic gastric mucormycosis were reported after use of naso-gastric tubes or even tongue depressors colonized by the fungus.[77] Other possible GI sites include terminal ileum,[78] and large bowel.[79] Mucor can infect other parts of the digestive system including liver,[80] bile duct.[81] It can be severe enough to involve many adjacent organs including pancreas.[82]

Cutaneous mucormycosis: Intact skin forms a barrier against mucor penetration. Cutaneous zygomycosis takes hold when this barrier is disrupted. This disruption can be caused by skin maceration, burns[83] or trauma.[84] It usually carries a better prognosis than other forms of mucormycosis until the fungal reaches deeper into muscle, bone or fascia where it causes severe necrosis; the mortality rate then becomes very high.[85]

Disseminated zygomycosis: This is the form that has the worse prognosis. Its mortality rate approaches 100%. Since mucor is an angiotropic fungus, any prior form of this mycosis can cause severe fungemia in immune compromised individuals with subsequent hematogenous spread to many body organs including brain, heart, lungs, and kidneys among others. Moreover, disseminated mucormycosis was described with intravenous drug use[86] and diabetics utilizing self-monitoring material.[87] Antemortem diagnosis can be very challenging. Blood cultures, in these severely ill patients, are usually negative. The diagnosis is suspected in the presence of disseminated organ infarction and necrosis.[88] Cases of endocarditis[89] and myocarditis[90] were observed.

Miscellaneous Mucormycosis: infecting agents from the mucorales order can infect any part of the body. Indeed, documented cases of this fungal infection were reported in the ear,[91] or limited to the parotid gland,[92] or in the intravascular system where it can cause severe micro-aneurysms or migratory thrombi,[93–94] adjacent to the spinal cord,[95] inside joints like the knee,[96] or affecting whole upper or lower limbs,[97] within the urinary tract,[98] and genital organs and pelvic floor.[99–100] The consequences can be disastrous regardless of the affected site.

## DIAGNOSIS

Diagnosing invasive zygomycosis is not an easy task. Its clinical picture can be extremely variable. Further, because of its relative rarity, it is usually missed in its early stage when the chances of cure are still reasonable. Unfortunately, close to half of phycomycosis cases are diagnosed post-mortem.[101] A high index of suspicion should always be kept when facing these variable presentations of mucormycosis especially when dealing with immune-compromised patients. When this infection is suspected, physicians have nowadays an extensive armamentarium at their disposition to try to pin down its elusive diagnosis. Initial blood work can reveal non-specific findings such as leukocytosis, hyperglycemia or acidosis. Frequently, stigmata of immunosuppression, including neutropenia, are encountered. Blood cultures are usually negative but, exceptionally, fungal growth in the blood can be observed.[102] Until now, there is no specific serologic test for mucormycosis. Radiological investigations, such plain x-rays or computed tomography, can be completely normal or demonstrate variable abnormal findings depending on the infection size and location. That location will guide the clinician to use further diagnostic tools in order to further clarify the diagnosis. Examples of such tools include bronchoscopy with bronchoalveolar lavage in case of pulmonary disease, upper or lower endoscopy for gastric or bowel lesions, or video assisted device for abdominal or thoracic infection.[103] However, obtaining tissue biopsy remains the gold standard for diagnosing invasive zygomycosis. Indeed, clinicians should not hesitate to obtain a good sample of the infected tissue as soon as possible to clinch the diagnosis. This task is relatively easy in cutaneous and rhinomaxillary mucormycosis; it becomes more challenging with deeper forms. Histopathology will reveal irregular broad non-septate hyphae and spores pathognomonic of mucor; with evidence of surrounding neutrophilic infiltration, necrosis and vasculature invasion.

## TREATMENT

Most medical textbooks and literature emphasizes three important cornerstones in the treatment of invasive zygomycosis. They are: reversal of the underlying condition(s), medical therapy and surgical debridement. In this review, and in addition to these necessary interventions, we would like to emphasize two more adjunctive principles, very much needed by patients who survive this horrible infection. They include: psychological support and physical rehabilitation.

1. Reversal of underlying condition(s): any predisposing factor, such as hyperglycemia or acidosis or malnutrition or immunosuppression, must be corrected if possible. This easy initial intervention improves the chances of survival.[104]
2. Medical therapy: until recently, the natural course of mucormycosis was usually fatal. A breakthrough in the treatment of deep mycoses occurred in 1953 when Drs Charles Smith and William Winn discovered amphotericin B from a soil isolate brought from the Orinoco Basin in Venezuela.[105] This discovery opened doors to the parenteral therapy for such mycoses including histoplasmosis, cryptococcosis, and mucormycosis. The first case of cure from this severe disease was reported by Harris in 1955.[9] There are two types of antifungal treatment for invasive zygomycosis:
  - Standard therapy: amphtericin B is a polyene macrolide. It continues to play a major role in the treatment of invasive zygomycosis. Both conventional and liposomal amphotericin B are effective against it; the liposomal form offers less infusion site side effects and milder nephrotoxicity, however, it generally costs more.[106] The duration of therapy varies from weeks to months depending on the site and severity of the infection.
  - Experimental therapy: newer antifungal medications are being currently developed. The orally administered posaconazole, from the family of azoles, recently showed promising results against the mucorales species.[107] Iron chelation is a novel adjunctive therapy that has potential role in the treatment of mucormycosis.[108] Future immunotherapy will probably hold some key answers in the management of zygomycosis.
3. Surgical intervention: surgical debridement is another cornerstone in treating invasive zygomycosis. It is usually extensive and can be disfiguring. It has to be done in earnest in addition to other therapeutic interventions.
4. Psychological support: patients infected with invasive mucomycosis face many difficult challenges. This infection can be prolonged and exhausting, it adds to the heavy burden(s) of their uncontrolled chronic condition, such as diabetes and its complications, immunosuppression from malignancy or AIDS, etc. These patients are clearly prone to psychological setbacks and major depression. Health care providers must pay special attention to these possibilities and provide necessary supportive and therapeutic treatment.
5. Physical rehabilitation: if the infected person with invasive zygomycosis escapes death he could carry severe stigmata, from the infection or its treatment, such as disfigurement, partial/complete loss of an extremity or organ function. In many instances, these patients need prolonged course of physical or occupational rehabilitation depending on the degree of their disability.

## CONCLUSIONS

Rare compared to other fungal infections, invasive zygomycosis is apparently gaining more ground. It is seen primarily in the immune-compromised patients. It has multiple clinical and radiologic presentations. Health care providers are urged to have a high degree of suspicion for it. Early tissue diagnosis and aggressive therapeutic intervention must be carried out as soon as possible. Psychological support and physical rehabilitation must be considered and provided for surviving patients. Unfortunately, even with early and aggressive intervention, invasive mucormycosis still carries poor prognosis.
